# Multi-Focus Image Fusion Method Based on Multi-Scale Decomposition of Information Complementary

**DOI:** 10.3390/e23101362

**Published:** 2021-10-19

**Authors:** Hui Wan, Xianlun Tang, Zhiqin Zhu, Weisheng Li

**Affiliations:** 1 College of Computer Science and Technology, Chongqing University of Posts and Telecommunications, Chongqing 400065, China; kyc3@cqnu.edu.cn (H.W.); liws@cqupt.edu.cn (W.L.); 2 College of Computer and Information Science, Chongqing Normal University, Chongqing 401331, China; 3 College of Automation, Chongqing University of Posts and Telecommunications, Chongqing 400065, China; zhuzq@cqupt.edu.cn

**Keywords:** multi-focus image fusion, singular value decomposition, multi-scale decomposition, PA-PCNN, quaternion, joint bilateral filter

## Abstract

Multi-focus image fusion is an important method used to combine the focused parts from source multi-focus images into a single full-focus image. Currently, to address the problem of multi-focus image fusion, the key is on how to accurately detect the focus regions, especially when the source images captured by cameras produce anisotropic blur and unregistration. This paper proposes a new multi-focus image fusion method based on the multi-scale decomposition of complementary information. Firstly, this method uses two groups of large-scale and small-scale decomposition schemes that are structurally complementary, to perform two-scale double-layer singular value decomposition of the image separately and obtain low-frequency and high-frequency components. Then, the low-frequency components are fused by a rule that integrates image local energy with edge energy. The high-frequency components are fused by the parameter-adaptive pulse-coupled neural network model (PA-PCNN), and according to the feature information contained in each decomposition layer of the high-frequency components, different detailed features are selected as the external stimulus input of the PA-PCNN. Finally, according to the two-scale decomposition of the source image that is structure complementary, and the fusion of high and low frequency components, two initial decision maps with complementary information are obtained. By refining the initial decision graph, the final fusion decision map is obtained to complete the image fusion. In addition, the proposed method is compared with 10 state-of-the-art approaches to verify its effectiveness. The experimental results show that the proposed method can more accurately distinguish the focused and non-focused areas in the case of image pre-registration and unregistration, and the subjective and objective evaluation indicators are slightly better than those of the existing methods.

## 1. Introduction

Due to the focal lengths of optical lenses, the images obtained by the camera include focused and defocused parts. Focused parts are sharper in the image, while defocused parts appear blurry. In order to obtain full-focus images, a common solution is utilizing multi-focus image fusion technology, to combine the focused parts of different images in the same scene. The combined full-focus image contains global clarity and rich details, and is more suitable for visual perception and computer processing. As an important branch of image fusion, multi-focus image fusion can be studied on three different levels, i.e., pixel level, feature level, and decision level [1]. Compared with the other two levels, pixel-level image fusion can maximally reserve the original information in the source image, giving it an edge over the other two in accuracy and robustness. Accordingly, it has become a common fusion method for multi-focus images. The method proposed in this paper is based on pixel-level multi-focus image fusion.

Multi-scale decomposition (MSD) is a technique usually applied in pixel-level multi-focus image fusion, and it was proven to be a very useful image analysis tool. The MSD-based fusion method can extract image feature information on different scales for image fusion. The mechanism of the MSD fusion method is as follows. Firstly, the source images are decomposed into multi-scale spaces by MSD, where there is one approximate component containing contours and several detail components storing salient features. Then, the decomposed coefficients of all scale spaces are fused, following the designed fusion strategies. Finally, the inverse multi-scale decomposed is used to reconstruct the final fused image. Undoubtedly, the choice of the multi-scale decomposition method and fusion strategy are two important factors of image fusion.

The selection of the multi-scale decomposition method needs to factor in the following aspects: firstly, the desirable feature extraction performance. One advantage of the MSD-based method is to separate spatially overlapping features in scales. Secondly, the decomposition algorithm. In practical applications, the execution efficiency of the algorithm is a key indicator. Finally, good generalization. It means being able to handle various types of images, including anisotropic blur and unregistration.

Since the 1980s and 1990s, various multi-scale decomposition methods have been applied in multi-focus image fusion [2], mainly containing Laplacian pyramid (LP) [3], gradient pyramid (GP) [3], discrete wavelet transform (DWT) [4], and so on. Although, DWT improves computational efficiency compared with LP and GP, it does not reflect shift invariance and direction selectivity, which undermine feature extraction. To address these problems, the dual-tree complex wavelet (DTCWT) [5] is proposed, which has shift invariance and direction selectivity, and is successfully applied for multi-focus image fusion [6]. Compared with the pyramid and wavelet transform, the multi-scale geometric analysis (MGA) [7,8,9] methods better reflect the inherent geometric structure of the image, and outperform in feature extraction, but the calculation is more complex and time-consuming.

In recent years, scholars have proposed new and efficient multi-scale decomposition methods, which show good performance in multi-focus image fusion. Typical methods include the following: Li et al. [10] proposed a two-scale decomposition method for multi-focus fusion with the guided filtering technique. Through simple average filtering, each source image is decomposed into a basic layer with large-scale variations and a detail layer containing small-scale details. The method is superior to many traditional MSD-based methods in terms of fusion performance and computational efficiency. Xiao et al. [11] proposed that the multi-scale hessian matrix can decompose the source images into small-scale feature components and large-scale background components, and effectively remove the pseudo-edges, which are introduced by image blurring and unregistered. The method shows good feature extraction and generalization capabilities. Zhang et al. [12] proposed a multi-scale decomposition scheme by changing the size of the structural elements, and extracting the morphological gradient information of the image on different scales to achieve multi-focus image fusion. The method shows the best execution efficiency. NaiduIn et al. [13] and Wan et al. [14] proposed multi-scale analysis and singular value decomposition are combined to perform multi-focus image fusion. This method achieves the stability and orthogonality equivalent of that achieved by SVD. Since no convolution operations are required, the fast decomposition speed means high execution efficiency of the algorithm.

In addition to developing novel methods for MSD, fusion rules also play a key role in image fusion. Advanced fusion rules and MSD methods form a complementary whole in image fusion, which promotes fusion performance. The fusion rules of multi-focus images are usually designed based on the focus measure between pixels. Commonly used focus measures incorporate spatial frequency (SF) [15], sum-modified-Laplacian (SML) [16], standard deviation (STD) [17], energy of gradient (EOG) [18], etc. Generally, simple pixel-based fusion rules are insensitive to anisotropic blur and misregistration. For example, fusion rules, such as direct comparison of decomposition coefficients and weighted average values based on spatial context. To improve fusion results, some complex fusion rules are proposed. Among these are block-based and area-based methods [19,20]. Firstly, the original images are divided into a number of blocks or regions. Then, the focus level and sharpness of each block or region is measured by image intensity. Finally, a block or region with a higher degree of focus as part of the fusion image is selected. However, the quality of image fusion depends on the selection of the image block sizes or the segmentation algorithms. When the image block is not selected correctly or the segmentation algorithm cannot correctly segment the area, the focus area cannot be correctly determined and extracted, and the boundary between the focus and the defocus area is prone to blur. Zhou et al. [21] proposed a new focus measure fusion method based on a multi-scale structure, which uses large-scale and small-scale focus measures to determine the clear focus area and weight map of the transition area, respectively. This method can reduce the influence of anisotropic blur and unregistration on image fusion. However, the transition area is artificially set and cannot accurately reflect the focus of the boundary. Ma et al. [22] proposed a random walk-based with two-scale focus measure for multi-focus image fusion. The method estimates a focus map directly from the two-scale imperfect observations obtained using small and large-scale focus measures. Since the random walk algorithm is used to model the estimation from the perspective of probability, this method is relatively time-consuming. In addition to the commonly used linear model fusion rules mentioned above, there are also some fusion rules based on non-linear methods. Dong et al. [23] proposed a multi-focus image fusion scheme by memristor-based PCNN. Hao et al. [24] review the state-of-the-art on the use of deep learning in various types of image fusion scenarios. The Generative Adversarial Network (GANS) proposed by Guo et al. [25] has also been successfully applied to multi-focus image fusion. When it comes to the deep learning model of multi-focus image fusion, the measurement of pixel activity level is obtained through the model. However, the difficulties in training a large number of parameters and large datasets have directly affected the image fusion efficiency and quality. Compared with deep learning methods, the conventional fusion methods are more extensible and repeatable, facilitating real-world applications. Thus, the paper mainly aims to improve the conventional multi-focus image fusion algorithms.

According to the above analyses, decomposition schemes and focus measures involved in the fusion strategy play important roles in multi-focus image fusion. In recent years, many novel algorithms have been proposed to improve the image fusion quality, but some existing problems still need to be addressed. Firstly, due to the diversity of fused images, the contour and detailed information of images cannot be fully expressed when images are decomposed by fixed basis and filter functions. Secondly, the boundary between the focused and defocused areas of the image gives rise to false edges, mainly due to the fact that the boundary between the two areas are not clearly distinguished, or that the two images are not registered. Finally, the artifacts are easily generated between the focused and unfocused flat regions, since the image details in those regions are extremely scanty [11].

In order to solve the problems, a novel multi-focus image fusion method based on multi-scale singular value decomposition (MSVD) is proposed in this paper. The method obtains low-frequency and high-frequency components with complementary information through two groups of double-layer decompositions with complementary structures and scales, and these components contain rich image structure and detailed information. The proposed fusion rules are then applied to fuse each component to obtain the final fusion image. Concretely, different fusion strategies and focusing measures are used to fuse the high-frequency and low-frequency sub-images, respectively, and two initial decision diagrams with complementary information are obtained. Hence, a definite focus area and a non-definite focus area are obtained. After that, the non-definite focus area is refined and transformed into a definite focus area, and the final decision map to complete the image fusion is obtained. The proposed method has the following advantages. Firstly, two groups of decomposition schemes with complementary structure and scale are designed to accurately obtain the focus of the boundary. Secondly, the proposed method combines multi-scale analysis and singular value decom-position for multi-focus image fusion. Singular value decomposition diagonalizes the image matrix according to the size of eigenvalues, so there is no redundancy between the decomposed images, which is suitable for different fusion rules for each component. Finally, by exploiting the image feature information contained in each decomposition layer of low-frequency components and high-frequency components, selecting different focus measures can better extract the image feature information.

Compared with the existing multi-focus image fusion method, the main innovations of the proposed method are as follows:

- The paper uses MSVD decomposition with a complementary structure and size for the first time, enhances the complementarity of the extracted image feature information and improves the ability to detect the focus area, in order to fully extract the structure and detailed information of the image.
- To fully extract the structure and details of the image, the complementary features extracted by different focus measures are developed as the external stimulus input of the PA-PCNN.
- Experiments are performed to verify the efficiency of the proposed method. The results show that the proposed method can effectively eliminate the pseudo edges caused by anisotropic blur or unregistration.

The structure of this paper is organized as follows. Section 2 proposes the multi-focus image fusion model based on multi-scale decomposition of information complementary. Section 3 analyses and discusses the results of the comparison with the latest methods. Finally, conclusions for this paper are provided in Section 4.

## 2. Proposed Multi-Focus Image Fusion Algorithm

Due to object displacement or camera shake during image acquisition, multi-focus images will produce unregistration and anisotropic blur. These factors can lead to erroneous focus judgment in the focus map obtained by the focus measure (FM), which make the fusion image appear blurred and distorted. In order to solve the above problems, Zhou et al. [21] proposed a two-scale fusion scheme. A large scale can better reduce blur and unregistration, and a small scale can better retain some details, so that the Halo effect of the fused image can be mitigated. However, this algorithm calculates its saliency map based on the covariance matrix of the region, and the fusion effect is not good for images without obvious edges or corners. In addition, an unknown area is defined near the boundary pixels of the focus area, and its width is set as $4\delta_{1}$. This artificially set unknown area cannot accurately reflect the focus of the boundary and will affect fusion. In response to the above problems, we propose a multi-focus image decomposition strategy based on a multi-scale singular value decomposition. In this strategy, two groups of low-frequency and high-frequency components with complementary information are obtained by two-level decomposition of the complementary structure and scale. According to the proposed fusion rules, each component is fused to obtain the final fusion image.

**Figure 1:**
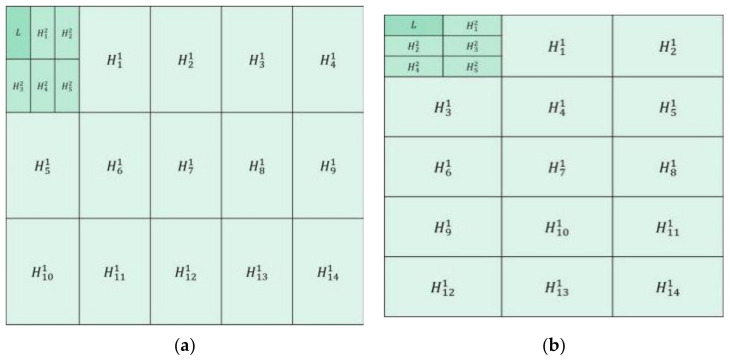
Two groups of double-layer multi-scale singular value decomposition schemes with complementary structure and scale. In the two decomposition schemes, $H_{1-14}^{1}$ are the high-frequency components of the first layer after decomposition, $H_{1-5}^{2}$ are the high-frequency components of the second layer after decomposition, *L* is the low-frequency component of the second layer after decomposition. (**a**) The first group of the decomposition scheme; (**b**) the second group of the decomposition scheme.

Figure 1a shows the first group of decomposition schemes. The first layer is to divide the source image into blocks in the size of 3 × 5 to achieve large-scale decomposition of the image. In the second layer, the low-frequency components obtained from the first layer are divided into blocks in the size of 2 × 3 to achieve small-scale image decomposition. Figure 1b shows the second group of decomposition schemes. The first layer is to divide the source image into blocks in the size of 5 × 3 to achieve the large-scale decomposition of the image. In the second layer, the low-frequency components obtained from the first layer are divided into blocks in the size of 3 × 2 to achieve mall-scale image decomposition (in Section 2.1.2 for details of image segmentation method).

**Figure 2:**
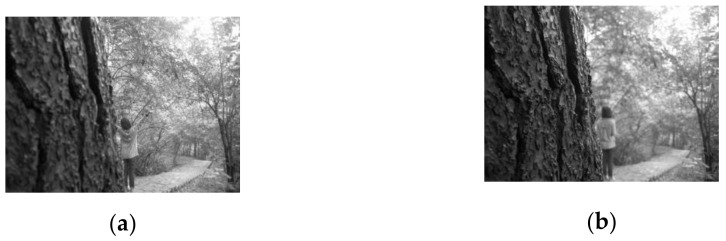

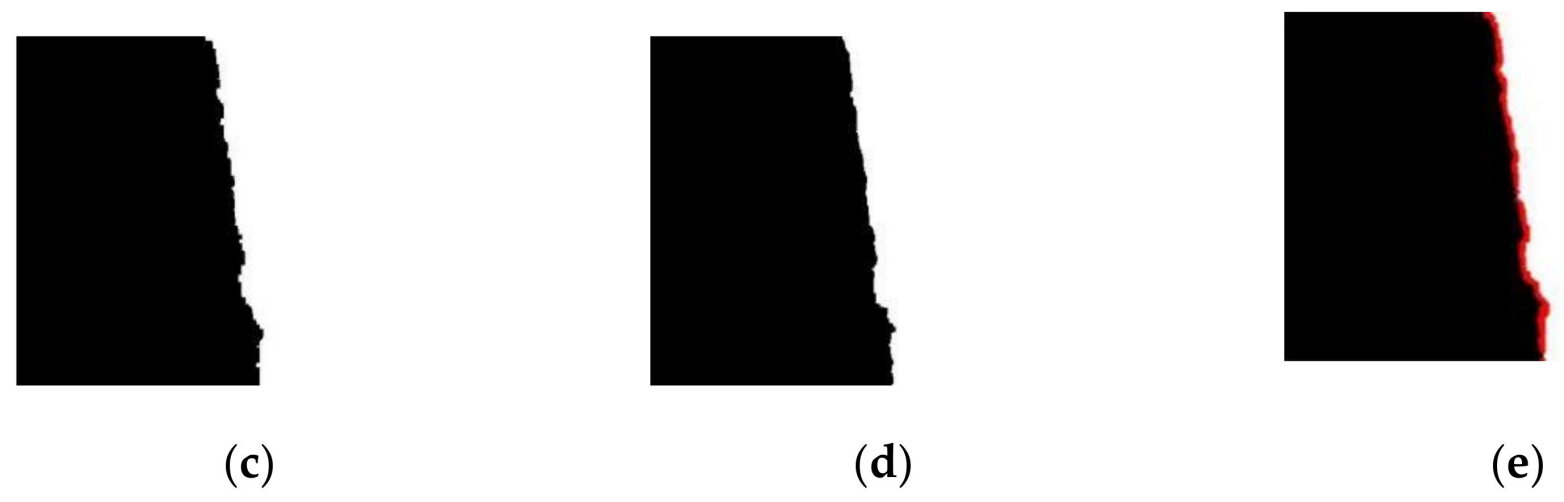
(**a**) the left focus source image; (**b**) the right focus source image; (**c**) fusion decision map obtained by the first group of the decomposition scheme; (**d**) fusion decision map obtained by the second group of the decomposition scheme; (**e**) the initial fusion decision map determined by (**c**,**d**), the black area corresponding to the decision value “0”, the white area corresponding to the decision value “1”, and the black and white areas are definite focus areas. The red area is the aliasing area of (**c**,**d**), which is the non-definite focus area.

The multi-scale decomposition scheme proposed in this paper uses block operation to achieve large-scale and small-scale decomposition of the image. Large-scale decomposition can better retain image structure information, and small-scale decomposition can better retain image detail information. Through the proposed fusion rule, the high and low frequency components obtained by the two decomposition schemes are fused, and two fusion decision maps with complementary information are obtained. These two fusion decision maps can make up for the poor fusion effect of images without giving rise to obvious edges and corners. It can also determine the blur region near the pixels of the focus region boundary. Figure 2 shows the two complementary information fusion decision maps obtained through the two decomposition schemes show in Figure 1 and the initial decision map determined through them. The initial decision map contains the definite focus area and the non-definite focus area.

### 2.1. Multi-Scale Singular Value Decomposition of Multi-Focus Image

#### 2.1.1. Multi-Scale Singular Value Decomposition

MSVD is an image decomposition method with simple calculations and is suitable for real-time applications. In image decomposition, it uses singular value decomposition (SVD) to perform a similar function to the FIR filter in wavelet transform, but MSVD is not like wavelet transform, which has a fixed set of basis vectors to decompose images, and its basis vectors depend on the image itself [13].

*X* is the matrix form of image f(x,y), $X\in R^{m\times n}$. When orthogonal matrixes $U\in R^{m\times m}$ and $V\in R^{n\times n}$ exist, we can obtain:

(1) $$U^{T}X\;V=\left[\begin{array}{ll} \Lambda_{r} & 0\\ 0 & 0 \end{array}\right]\;\equiv \Lambda \in R^{m\times n}$$

According to the transformation of Equation (1), the singular value decomposition of *X* can be obtained as:

(2) $$X=U\left[\begin{array}{ll} \Lambda_{r} & 0\\ 0 & 0 \end{array}\right]{\left(V\right)}^{T}$$

In Equation (2), $\Lambda_{r}=diag\{\lambda_{1},\lambda_{2},\cdots ,\lambda_{r}\},\lambda_{1}\geq \lambda_{2}\geq \cdots \geq \lambda_{r},$. *r* is the rank of the matrix *X*, $\lambda_{i}(1\leq i\leq r)$ is the singular value of *X*. The matrix singular value has strong stability, and will not change with image scaling and rotation. *U* and *V* are the eigenvectors corresponding to the singular values, and they depend on the image *X*. The amount of image information represented by eigenvector is positively related to the size of the corresponding singular value. The larger the singular value, the more image information it contains, which corresponds to the approximate part of the image. The smaller singular values correspond to the detailed parts of the image, which is the high frequency part of the image. Therefore, the image can be separated into approximate and detailed information according to the size of the singular values.

#### 2.1.2. Decomposition of Multi-Focus Image

In order to achieve multi-scale decomposition of the multi-focus images, they are divided into non-overlapping m×n blocks, and each sub-block is arranged into an *mn* × 1 vector. By combining these column vectors, a matrix $X^{\prime }$ with a size of $mn\times \left(MN/mn\right)$ can be obtained. The singular value decomposition of $X^{\prime }$ is:

(3) $$X^{\prime }=U^{\prime }\Lambda^{\prime }{\left(V^{\prime }\right)}^{T}$$

$U^{\prime }$ and $V^{\prime }$ are orthogonal matrices, according to Equation (3):

(4) $$S={\left(U^{\prime }\right)}^{T}X^{\prime }=\Lambda^{\prime }{\left(V^{\prime }\right)}^{T}$$

The size of the matrix *S* is $mn\times \left(MN/mn\right)$.

According to the singular value decomposition mentioned above, the first column vector of $U^{\prime }$ corresponds to the maximum singular value. When it is left multiplied by the matrix $X^{\prime }$, the first row S(1,:) of *S* carries the main information from the original image, which can be regarded as the approximate or smooth component of the original image. Similarly, the other row S(2:mn,:) of *S* corresponds to smaller singular values, which retains such detailed information as the texture and edges of the original image. Therefore, through singular value decomposition, the image can be decomposed into low-frequency and high-frequency subimages by the singular value to achieve the multi-scale decomposition of the image. The schematic diagram of the multi-focus image MSVD scheme proposed in this paper is illustrated in Figure 1. In order to clearly illustrate the image decomposition process, it is assumed that there is a source image with a size of 300 × 300. According to the decomposition scheme in Figure 1a and the above mentioned image decomposition steps, the source image is divided into blocks of size 3 × 5 to achieve the first-layer large-scale decomposition. After that, 1 low-frequency component and 14 high-frequency components are obtained, and the size of each component is 100 × 60. The second-layer of decomposition is to divide the low-frequency components of the first-layer into blocks of size 2 × 3 to achieve small-scale decomposition. Moreover, 1 low-frequency component and 5 high-frequency components are obtained, and the size of each component is 50 × 20. After fusion of the components, the final fusion image is acquired through the inverse MSVD transformation.

### 2.2. Low-Frequency Component Fusion

The low-frequency sub-image of the multi-focus image obtained by the MSVD decomposition scheme proposed in this paper reflects the overall characteristics of the image, and mainly contains contour and energy information. In this paper, we use the algebraic operations and spatial characteristics of quaternions to calculate the local energy of low-frequency components. Joint bilateral filter (JBF) is used to get the structure information of low-frequency components, combine the energy and structure information to calculate the weight to obtain the fusion decision map. The fused low-frequency components are obtained according to the decision map.

#### 2.2.1. Quaternion

Quaternions were first introduced in 1843 by British mathematician Hamilton [26]. They can be considered an extension of complex numbers. The general form of a quaternion is expressed as follows:

(5) Q=a+bi+cj+dk

where

$$\begin{array}{l} i^{2}=j^{2}=k^{2}=ijk=-1,\\ ij=-ji=k,jk=-kj=i,ki=-ik=j. \end{array}$$

and where *a* is the real part, *bi*, *cj*, and *dk* are three imaginary parts. If the real part *a* is zero, *Q* is called a pure quaternion.

The modulus of a quaternion is defined as:

(6) $$\left|Q\right|=\sqrt{QQ^{*}}=\sqrt{a^{2}+b^{2}+c^{2}+d^{2}}$$

where $Q^{*}$ is defined as the conjugate of the quaternion *Q*, $Q^{*}=a-bi-cj-dk$.

The unit vector of a quaternion *Q* is defined as:

(7) $$q=\frac{Q}{\left|Q\right|}$$

Define two quaternions as

$$Q_{1}(Q_{1}=a+q_{v1})\;\mathrm{and}\;Q_{2}(Q_{2}=b+q_{v2})$$

In Equation (8), quaternion multiplication can be represented using the cross and dot product.

(8) $$\begin{array}{ll} Q_{1}Q_{2} & =(a+q_{v1})(b+q_{v2})\\ & =(ab-q_{v1}.q_{v2})+(aq_{v2}+bq_{v1}+q_{v1}\times q_{v2}) \end{array}$$

where $q_{v1}$ and $q_{v2}$ are the vector parts of each quaternion. $q_{v1}\cdot q_{v2}$ and $q_{v1}\times q_{v2}$ represent the dot product and cross product of the two vectors, respectively.

#### 2.2.2. Joint Bilateral Filter

Bilateral filter (BF) is a nonlinear filtering method, which combines the spatial proximity and pixel value similarity of the image. BF can achieve edge preservation and denoising during image fusion. However, the weights of the bilateral filter are not stable enough, and the joint bilateral filter (JBF) introduces the guiding image on the basis of the bilateral filter, making the weights more stable. JBF can be expressed as follows:

(9) $$J(x)=\frac{1}{W}\sum_{y\in \Omega }G(x,y,\delta_{s})G(O(x),O(y),\delta_{r})I(y)$$

*W* is the regularization factor, defined as:

$$W=\sum_{y\in \Omega }G(x,y,\delta_{s})G(O(x),O(y),\delta_{r})$$

The Gaussian kernel function G is expressed as:

$$G(x,y,\delta )=e^{-\frac{{\left\|x-y\right\|}^{2}}{2\delta^{2}}}$$

In Equation (9), the set of adjacent pixels is denoted as Ω, $\delta_{s}$, and $\delta_{r}$ are the parameters of two Gaussian kernel functions, which are used to control the influence of Euclidean distance and pixel similarity. The Gaussian kernel function will attenuate as the distance between x and y increases. When the distance between *x* and *y* is less than $\delta_{s}$ or the difference between two pixel values is less than $\delta_{r}$, the pixel value I(y) of *y* has a greater impact on the value of J(x). Different from the bilateral filter, O(x) and O(y) are the guiding pixel values of *x* and *y*, respectively. The guiding image *O* can provide more reliable information for the structure of the output image and obtain a more optimized similarity Gaussian kernel weight.

#### 2.2.3. Low-Frequency Component Fusion Rule

The low-frequency component contains most of the energy and contour information of the image. Therefore, in the low-frequency fusion process, the energy and contour information of the image should be taken into account. In this paper, a new low-frequency component fusion method is proposed. Firstly, the local energy of low-frequency component is calculated using the neighborhood of pixels represented by quaternions. Secondly, we use JBF to get the edge contour information of the low frequency component. Then, we combine the local energy and the edge energy to calculate the weight of the low-frequency component to obtain the fusion decision map. Finally, the fused low-frequency component is obtained according to the decision map. The detailed fusion process is as follows:

1. Select the pixel in the 3 × 3 domain of the target pixel to construct quaternion $Q_{I}^{1},Q_{I}^{2}$, and calculate the local energy $E_{I}^{L}$ of the low-frequency component:
  (10) $$\begin{array}{l} E_{I}^{L}(x,y)\;=Q_{I}^{1}Q_{I}^{2}*f_{I}^{L}(x,y)\\ Q_{I}^{1}=f(x,y+1)+if(x,y-1)+jf(x-1,y)+kf(x+1,y)\\ Q_{I}^{2}=f(x+1,y+1)+if(x+1,y-1)+jf(x-1,y-1)+kf(x-1,y+1) \end{array}$$

In Equation (10), I=A,B, (x,y) represent the position of the low-frequency component pixel. $Q_{I}^{1}$ is the quaternion formed by the front, back, left, and right pixels in the neighborhood of pixel (x,y). $Q_{I}^{2}$ is the quaternion formed by diagonal pixels in the neighborhood of pixel (x,y). In the calculation of $E_{I}^{L}$, $Q_{I}^{1},Q_{I}^{2}$ is constructed as a unit vector according to Equation (7).

2. JBF is used to process the local energy map $E_{I}^{L}$ of low frequency components to get the energy map $S_{I}^{L}$ of edge pixels:
  (11) $$S_{I}^{L}=JBF(E_{I}^{L},f_{I}^{L},w,\delta_{s},\delta_{r})$$

In Equation (11), $E_{I}^{L}$ is the local energy of the low-frequency component, with low-frequency component $f_{I}^{L}$ as a guide map, w represents the local window radius, $\delta_{s}$ is the standard deviation of the spatial domain kernel, and $\delta_{r}$ the standard deviation of the range kernel.

3. According to the local energy $E_{I}^{L}$ and edge energy $S_{I}^{L}$ of the low-frequency component, the weight of the low-frequency component is calculated.
  (12) $$\begin{array}{l} d_{A}^{L}=\left\{\begin{array}{l} 1,E_{A}^{L}\cdot S_{A}^{L}\;\geq \;E_{B}^{L}\cdot S_{B}^{L},\\ 0,otherwise, \end{array}\right.\\ \;\;\;d_{B}^{L}=1-d_{A}^{L}. \end{array}$$
4. The fusion image of the low-frequency component is obtained by the following formula:
  (13) $$f_{F}^{L}=d_{A}^{L}\cdot f_{A}^{L}+d_{B}^{L}\cdot f_{B}^{L}$$

### 2.3. High-Frequency Component Fusion

The high frequency component corresponds to the sharply changing part of the image, including the texture, details, and edge information of the image, which impacts the clarity and visual effects of the fused image. Pulse coupled neural network (PCNN) is a simplified artificial neural network constructed by Eckhorn based on the cat’s eye vision principle. Its signal form and processing mechanism are more in line with the physiological characteristics of the human visual nervous system. In order to improve the quality of the fused image, this paper proposes to use an adaptive PCNN strategy to fuse high-frequency components. The first layer of image decomposition selects the local spatial frequency (SF) as the external stimulus input of the PCNN, and the second layer selects the local standard deviation (STD) as the external stimulus input of PCNN.

#### 2.3.1. PA-PCNN

PCNN can capture image edge and detailed information without any training process. It is a feedback single-layer network composed of several neurons connected with each other. It has three functional units: feedback input domain, connection input domain, and pulse generation domain. The traditional PCNN model needs to determine parameters, such as link strength, various amplitudes, and attenuation coefficients. In order to avoid the insufficiency of manually setting parameters, a simplified PCNN model [27,28] is proposed, which is described as follows:

(14) $$F_{ij}[n]=S_{ij}$$

(15) $$L_{ij}[n]=V_{L}\sum_{kl}W_{ijkl}Y_{kl}[n-1]$$

(16) $$U_{ij}[n]=e^{-a_{f}}U_{ij}[n-1]+F_{ij}[n](1+\beta L_{ij}[n])$$

(17) $$Y_{ij}[n]=\left\{\begin{array}{l} 1,\;if\;U_{ij}[n]>E_{ij}[n-1]\\ 0,\;otherwise \end{array}\right.$$

(18) $$E_{ij}[n]=e^{-a_{e}}E_{ij}[n-1]+V_{E}Y_{ij}[n]$$

$F_{ij}[n]$ and $L_{ij}[n]$ are the external stimulus input and link input of the pixel at position (*i*, *j*) during the nth iteration, and $S_{ij}$ is the input image. The parameter $V_{L}$ is the amplitude of the link input $L_{ij}[n]$, which controls $L_{ij}[n]$ together with $W_{ijkl}$ and $Y_{kl}[n-1]$, and $W_{ijkl}=\left[\begin{array}{ccc} 0.5 & 1 & 0.5\\ 1 & 0 & 1\\ 0.5 & 1 & 0.5 \end{array}\right]$ is the synaptic weight matrix. The internal activity item $U_{ij}[n]$ consists of two parts: the first part $e^{-a_{f}}U_{ij}[n-1]$ is the exponential decay part of the internal activity of the previous iteration, and $a_{f}$ is the exponential decay coefficient. The second part $F_{ij}[n](1+\beta L_{ij}[n])$ is the nonlinear modulation of $F_{ij}[n]$ and $L_{ij}[n]$, where the parameter β is the link strength. $Y_{ij}[n]$ depends on the current internal activity item $U_{ij}[n]$ and the dynamic threshold $E_{ij}[n-1]$ during the last iteration. When $U_{ij}[n]>E_{ij}[n-1]$, $Y_{ij}[n]=1$, PCNN is in an ignition state. By contrast, $Y_{ij}[n]=0$, PCNN is in an unfired state. $Y_{ij}[n]=0$ and $V_{E}$ are the exponential decay coefficient and amplitude of $E_{ij}[n]$, respectively. There are 5 free parameters in the parameter adaptive PCNN model: $a_{f},\beta ,V_{L},a_{e},V_{E}$. These parameters can be calculated by the following formula [27,28]:

(19) af=log1δ(s),βVL=SmaxS′−16

(20) $$V_{E}=e^{-a_{f}}+1+6\beta V_{L}$$

(21) $$a_{e}=\ln\frac{V_{E}}{S^{\prime }(\frac{1-e^{-3a_{f}}}{1-e^{-a_{f}}}+6\beta V_{L}e^{-a_{f}})}$$

**Figure 3:**
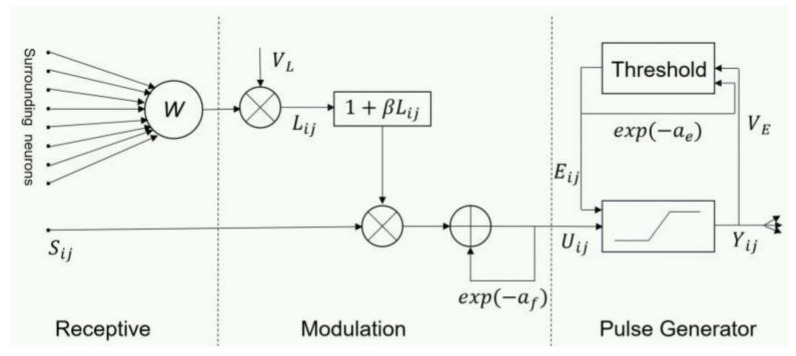
The diagram of a neuron in PA-PCNN model.

The smaller the value of $a_{f}$, the greater the dynamic range of $U_{ij}[n]$. δ(s) is the standard deviation of normalized image *S*. *β* and $V_{L}$ are the weights of $\beta V_{L}$, it can be regarded as a whole as the weighted link strength. The maximum intensity value *S*_max_ of the input image and the optimal histogram threshold $S^{\prime }$ jointly determine the value of $\beta V_{L}$. $\beta V_{L}$ and $a_{f}$ are combined to get $V_{E}$ and $a_{e}$. Figure 3 shows the PA-PCNN model used in the multi-focus image fusion method proposed in this paper.

#### 2.3.2. Space Frequency and Standard Deviation

The spatial frequency (SF) and standard deviation (STD) of an image are two important indicators of the details of the image.

Spatial frequency is defined as:

(22) $$\begin{array}{l} SF=\sqrt{RF^{2}+CF^{2}}\\ RF=\sqrt{\frac{1}{M\times N}\sum_{i=1}^{M}\sum_{j=2}^{N}{\left[f(i,j)-f(i,j-1)\right]}^{2}}\\ CF=\sqrt{\frac{1}{M\times N}\sum_{i=2}^{M}\sum_{j=1}^{N}{\left[f(i,j)-f(i-1,j)\right]}^{2}} \end{array}$$

*RF* is the row frequency and *CF* is the column frequency. The spatial frequency (SF) of the image indicates the clarity of the spatial details of the image.

Standard deviation is defined as:

(23) $$\begin{array}{l} STD=\sqrt{\frac{1}{M\times N}\sum_{i=1}^{M}\sum_{j=1}^{N}{\left[f(i,j)-\mu \right]}^{2}}\\ \mu =\frac{1}{M\times N}\sum_{i=1}^{M}\sum_{j=1}^{N}f(i,j) \end{array}$$

The image standard deviation represents the statistical distribution and contrast of the image. The larger the standard deviation, the more scattered the gray level distribution, the greater the contrast, and the more prominent the image details. *μ* is the mean value of the image.

Spatial frequency and standard deviation reflect the details of the image from different aspects, and the two indicators are complementary.

#### 2.3.3. High-Frequency Component Fusion Rule

The high-frequency components of the source image obtained through multi-scale and multi-layer decomposition contain important details of the image. As the number of decom-position layers increases, the detailed features of high-frequency components become more prominent. In order to make the image fusion effect better meet the physiological characteristics of the human visual nervous system, in the first layer and second layer decomposition of high-frequency components, local spatial frequency (SF) and local standard deviation (STD) are, respectively, selected as external stimulus inputs of PA-PCNN, and to achieve the fusion of high-frequency components. The fusion procedure of high-frequency components is as follows:

1. In the first layer of decomposition, SF is used as the external stimulus input of PA-PCNN, and the number of ignitions of high-frequency components is obtained by
  (24) $$T_{S}^{1}[n]=T_{S}^{1}[n-1]+Y_{S}^{1}[n],(S=A,B)$$
2. Weight coefficient of high-frequency components is obtained by:
  (25) $$\begin{array}{l} d_{A}^{H1}=\left\{\begin{array}{l} 1,\;if\;T_{A}^{1}[n]\;>T_{B}^{1}[n],\;\\ 0,\;otherwise, \end{array}\right.\\ \;\;\;d_{B}^{H1}=1-d_{A}^{H1}. \end{array}$$
3. High-frequency components after fusion is obtained by:
  (26) $$f_{F}^{H1}=d_{A}^{H1}\cdot f_{A}^{H1}+d_{B}^{H1}\cdot f_{B}^{H1}$$

In the same way, STD is used as the external stimulus input of PA-PCNN to obtain the fused high-frequency components of the second layer decomposition.

(27) $$f_{F}^{H2}=d_{A}^{H2}\cdot f_{A}^{H2}+d_{B}^{H2}\cdot f_{B}^{H2}$$

*H*1 represents the high-frequency component decomposed in the first layer, and *H*2 represents the high-frequency component decomposed in the second.

### 2.4. Non-Definite Focus Region Fusion

A multi-focus image fusion method is commonly used to obtain the final fusion image based on the decision maps. However, the decision maps are often inaccurate, especially at the boundary between the focus and defocus regions. To better determine the focus attribute of the boundary, we propose to define the aliasing region of the two complementary initial decision graph boundaries as the undetermined focus region (the red region in Figure 2e). On this basis, the measurement method combining local spatial frequency (SF) and local standard deviation (STD) (Section 2.3.2) is used to convert the non-definite focus region into a definite focus region, and accurate fusion decision map is obtained, and can effectively address an out-of-focus blur caused by anisotropic blur and unregistration. The specific fusion process is as follows:

1. Based on the two complementary decision maps, an initial decision map *D_F_* containing the definite focus region and the non-definite focus region is obtained.
  (28) $$\begin{array}{lll} D_{F}=(D_{1}+D_{2})\cdot /2 & \\ D_{F}\left(i,j\right)\in D_{Iden}, & & D_{F}\left(i,j\right)=1\;\mathrm{or}\;D_{F}\left(i,j\right)=0\\ D_{F}\left(i,j\right)\in D_{Uniden}, & & D_{F}\left(i,j\right)=0.5 \end{array}$$
  where *D*_1_ is the fusion decision map obtained by the first group of decomposition scheme (Figure 2c), *D*_2_ is the fusion decision map obtained by the second group of the decomposition scheme (Figure 2d), *D_F_* is the initial decision map (Figure 2e). When $D_{F}(i,j)=1$ or $D_{F}(i,j)=0$, $D_{F}(i,j)$ belongs to the definite focus region *D_Iden_*; when $D_{F}(i,j)=0.5$, $D_{F}(i,j)$ belongs to the definite focus region *D_Uniden_* (the red region in Figure 2e).
2. The weight coefficient of the non-definite focus region is calculated by
  (29) $$Q_{U}^{Uniden}=\frac{1}{m\times n}\sum_{m=0}^{w}\sum_{n=0}^{w}\left(SF_{U}(i+m,j+n)*STD_{U}(i+m,j+n\right)),U=(A\;or\;B)$$
  (30) $$\begin{array}{cc} d_{A}^{Uniden}= & \left\{\begin{array}{ll} 1, & if\;Q_{A}^{Uniden}\cdot f_{A}^{Uniden}>Q_{B}^{Uniden}\cdot f_{B}^{Uniden},\\ 0, & otherwise, \end{array}\right.\\ & d_{B}^{Uniden}=1-d_{A}^{Uniden}. \end{array}$$
3. The non-definite focus region fusion is calculated by
  (31) $$f_{F}^{Uniden}=d_{A}^{Uniden}ʷf_{A}^{Uniden}+d_{B}^{Uniden}\cdot f_{B}^{Uniden}$$
  where $f_{A}^{Uniden}$ and $f_{B}^{Uniden}$ are non-definite focus regions of the source multi-focus images.

### 2.5. The Proposed Multi-Focus Image Fusion Method

Step 1: the two-layer MSVD decomposition with the complementary structure and scale (Figure 1) is performed on two multi-focus images, A and B, respectively, and two groups of information complementary low-frequency components and high-frequency components are obtained. In each group of decomposition, the source image is decomposed into a low-frequency component *L* and multiple high-frequency components $H_{i}^{c}$.

Step 2: different fusion rules are used to fuse the low-frequency components *L* and high-frequency components $H_{i}^{c}$ respectively, and the information complementary decision map *D*_1_ and *D*_2_ are obtained.

Step 3: the complementary decision maps in Step 2 are exploited, and the initial decision map *D_F_* containing the definite focus region and the non-definite focus region is obtained. The non-definite focus region *D_Uniden_* in *D_F_* is the aliasing area at the boundary of the complementary decision maps. With the adoption of the proposed focus measurement method (in Section 2.4), the non-definite focus region *D_Uniden_* is transformed into the definite focus region, and the final fusion decision map *D_FF_* is obtained.

Step 4: according to the fusion decision map *D_FF_* obtained in Step 3, the final fusion image is obtained.

**Figure 4:**
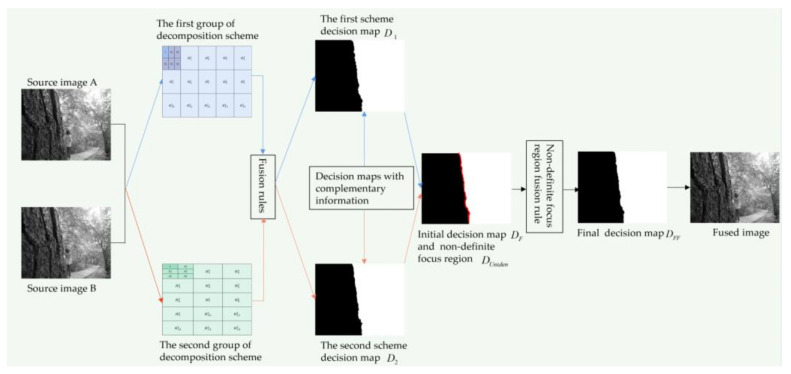
Schematic diagram of the proposed method of multi-focus image fusion. *D*_1_ is the first scheme decision map; *D*_2_ is the second scheme decision map; the red region in the initial decision map *D_F_* is the non-definite focus region *D_Uniden_*. $H_{i}^{c}$ is the *i*-th high-frequency component in the *c*-th layer decom-position, where *c* = 1 or 2.

Figure 4 illustrates the principle diagram of the method in this paper, which corresponds to the above fusion steps.

## 3. Experiments and Discussion

In order to verify the effectiveness of the proposed method, we first compare the proposed method with some classic and state-of-the-art methods, which are fusion methods based on traditional ideas. They are the curvelet transform (CVT) [29], the singular value decomposition in discrete cosine transform (DCT_SVD) [30], the dual-tree complex wavelet transform (DTCWT) [5,29], the image matting for fusion of multi-focus images (IFM) [31], the Laplacian pyramid (LP) [29], the multi-resolution singular value decomposition (MSVD) [13], the multi-scale weighted gradient-based fusion (MWGF) [21], the nonsubsampled contourlet transform (NSCT) [29,32]. The codes for the eight methods for comparison are provided by the authors of the corresponding papers, the MATLAB programs are all available online, and the parameters are the default values presented in the original papers. In addition, we select 13 pairs of multi-focus images commonly used in image fusion for comparative experiments, where 6 pairs of source images are provided by Lu et al. [1], and 4 pairs of source images are provided by Zhang et al. [33], and 3 other pairs of source images are obtained from the website [34]. In order to verify the performance of the proposed method, unregistered and pre-registered multi-focus images are specially selected for experimental analyses. Then, the proposed method is also compared with FuseGAN and CNN [25] methods, which are related to deep learning. The data sets, objective metrics, and fusion results used in the FuseGAN and CNN all derive from [25]. Finally, an ablation experiment is also carried out to test the effect of eliminating the PCNN method from the fusion result.

The decomposition parameters setting of the proposed method are: in the first group, the first layer is divided into 3 × 5 blocks, and the second layer is divided into 2 × 3 blocks; in the second group, the first layer is divided into 5 × 3 blocks, and the second layer is divided into 3 × 2 blocks (in Section 2.1.2 and Figure 1 for details of the parameters setting).

### 3.1. Comparative Analysis of Fusion Results Based on Traditional Methods

#### 3.1.1. Subjective Analysis of Pre-Registered Image Fusion Results

**Figure 5:**
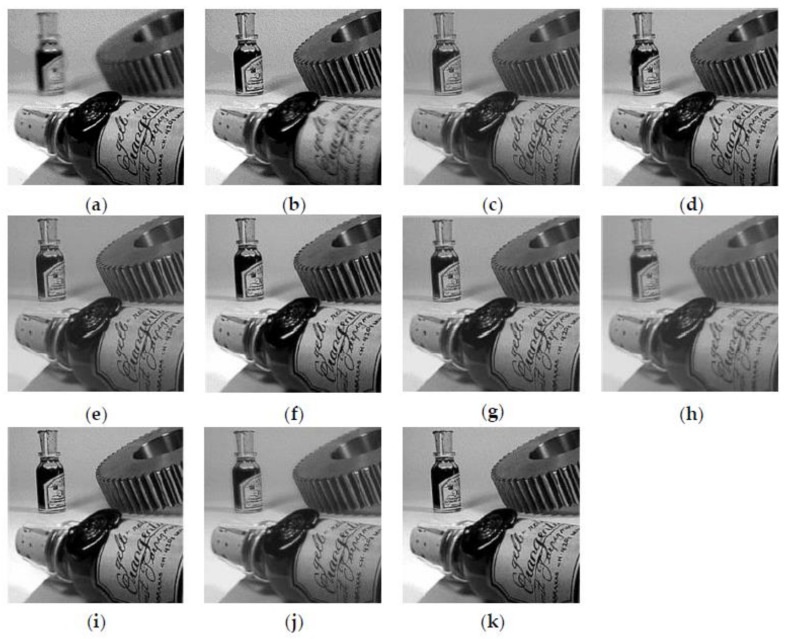
The source images of “wine” and the fusion results of different methods. (**a**) Source image A, (**b**) source image B, (**c**) curvelet, (**d**) DCT_SVD, (**e**) DTCWT, (**f**) IFM, (**g**) LP, (**h**) MSVD, (**i**) MWGF, (**j**) NSCT, (**k**) proposed method.

**Figure 6:**
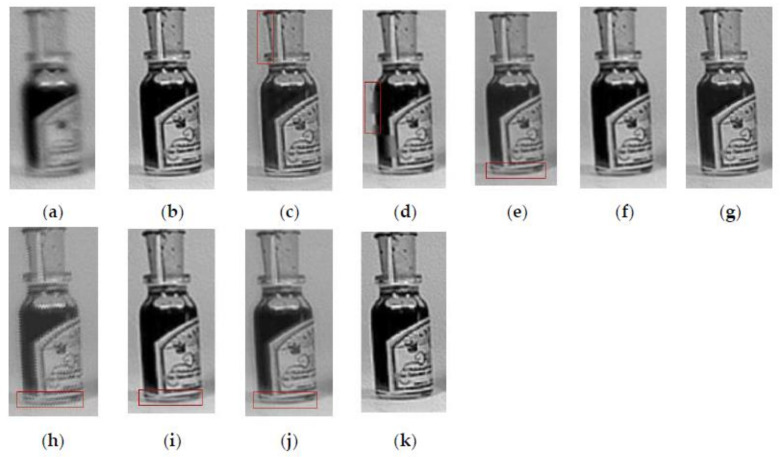
The partial enlarged regions taken from Figure 5a–k. (**a**) Source image A, (**b**) source image B, (**c**) curvelet, (**d**) DCT_SVD (**e**) DTCWT, (**f**) IFM, (**g**) LP, (**h**) MSVD, (**i**) MWGF, (**j**) NSCT, (**k**) proposed method.

**Figure 7:**
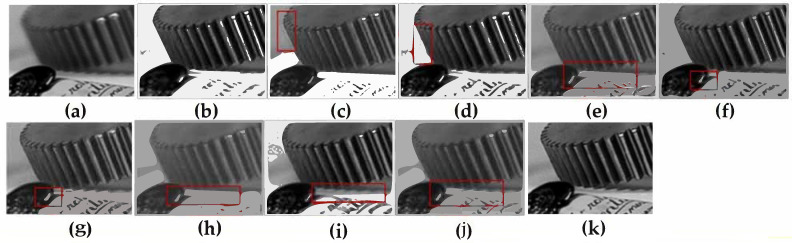
The partial enlarged regions taken from Figure 5a–k. (**a**) Source image A, (**b**) source image B, (**c**) curvelet, (**d**) DCT_SVD (**e**) DTCWT, (**f**) IFM, (**g**) LP, (**h**) MSVD, (**i**) MWGF, (**j**) NSCT, (**k**) proposed method.

**Figure 8:**
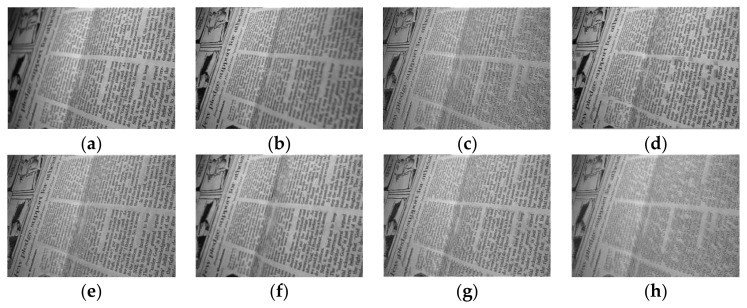

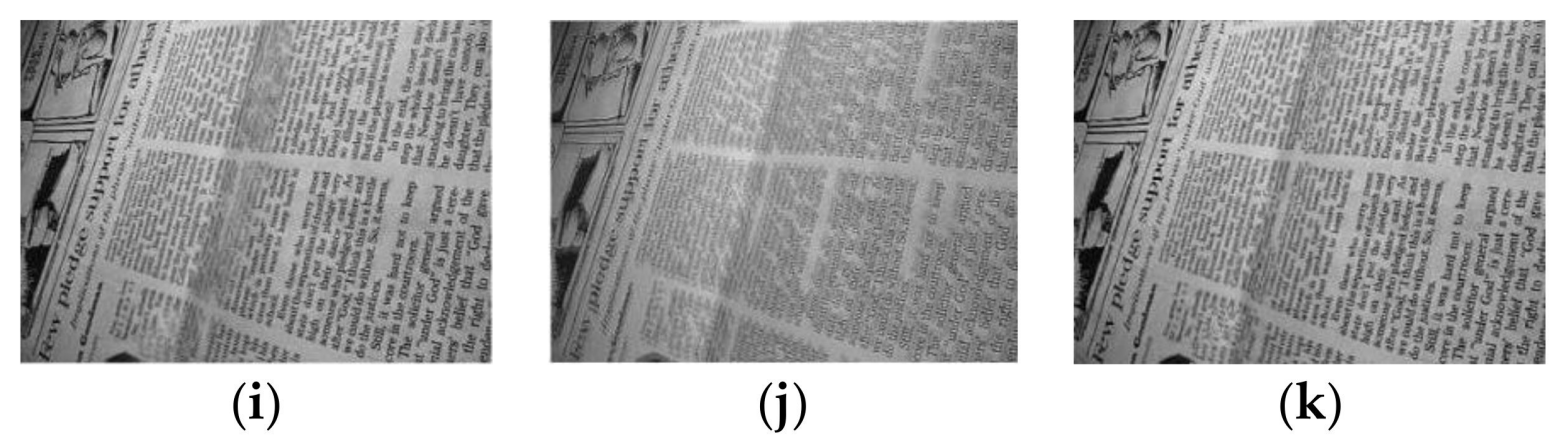
The source image of “newspaper” and the fusion results of different methods. (**a**) Source image A, (**b**) source image B, (**c**) curvelet, (**d**) DCT_SVD (**e**) DTCWT, (**f**) IFM, (**g**) LP, (**h**) MSVD, (**i**) MWGF, (**j**) NSCT, (**k**) proposed method.

**Figure 9:**
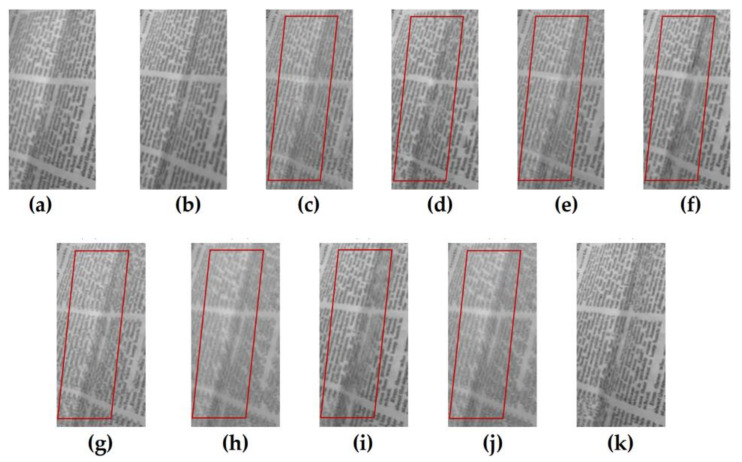
The partial enlarged regions taken from Figure 8a–k. (**a**) Source image A, (**b**) source image B, (**c**) curvelet, (**d**) DCT_SVD (**e**) DTCWT, (**f**) IFM, (**g**) LP, (**h**) MSVD, (**i**) MWGF, (**j**) NSCT, (**k**) proposed method.

Figure 5 shows the fusion results of the “wine” source image obtained by different multi-focus image fusion methods. Figure 5a,b are the source images of the front focus and the back focus, respectively. Figure 5c–j are the fusion results obtained by the curvelet, DCT_SVD, DTCWT, IFM, LP, MSVD, MWGF, and NSCT methods. Figure 5k is the fusion results achieved by the proposed method. Figure 6 and Figure 7 are enlarged regions of the local details of Figure 5. In Figure 6, the part marked by the red frame shows that the fused image is introduced; the artefacts and blurred edges are produced by the fusion method of curvelet, DCT_SVD, DTCWT, MSVD, MWGF, and NSCT, respectively. In Figure 7, the red regions near the gear also produce the pseudo-edges, and are generated by curvelet, DCT_SVD, DTCWT, IFM, LP, MSVD, MWGF, and NSCT. It is found that the proposed method achieves the best fusion results among these methods. Figure 8 shows the fusion results of the “newspaper” source images obtained by different fusion methods. Figure 8a,b are two source images of the left focus image and the right focus image, respectively. Figure 8c–j are the fusion comparative results of the eight methods, and (k) is the fusion result of the proposed method. Figure 9 presents the local detail magnified regions of Figure 8. The red regions are the boundaries between the focus regions and the defocus regions. The fusion result suggests the proposed method is clearer at the boundary, and that the characteristics of the source image are better preserved than other methods, whose fusion results have blurred edges and artifacts.

#### 3.1.2. Subjective Analysis of Unregistered Images Fusion Results

**Figure 10:**
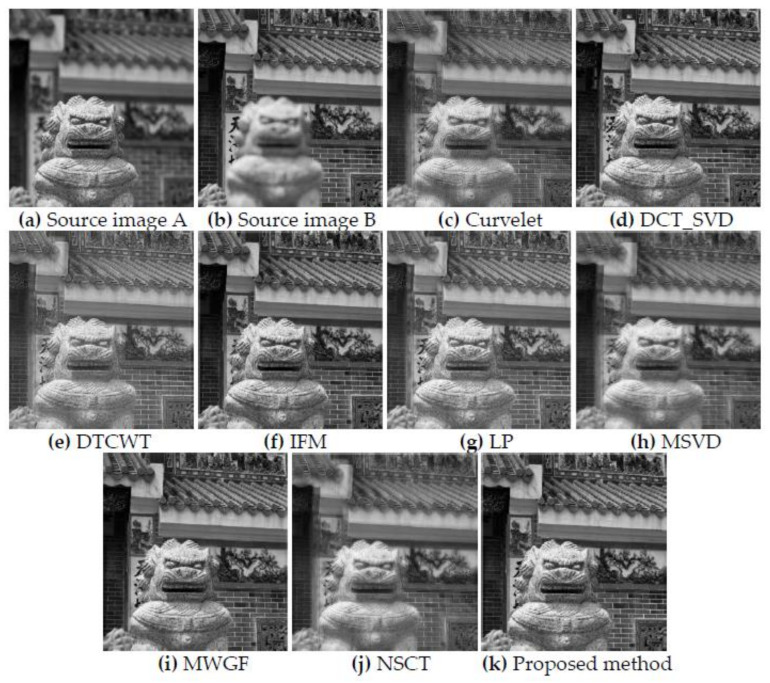
The source image of “temple” and the fusion results of different methods.

**Figure 11:**
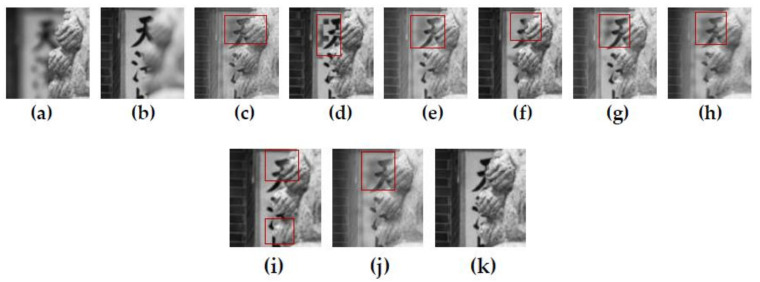
The partial enlarged regions taken from Figure 10a–k. (**a**) Source image A, (**b**) Source image B, (**c**) Curvelet, (**d**) DCT_SVD (**e**) DTCWT, (**f**) IFM, (**g**) LP, (**h**) MSVD, (**i**) MWGF (**j**) NSCT (**k**) Proposed method.

Figure 10 shows the fusion results of the “temple” source images obtained by nine different multi-focus image fusion methods. Figure 10a,b are two source images of the front focus image and the back focus image, respectively. From the stones in the lower left corners of the source images (a) and (b), it can be see that the two images have been displaced and have not been registered. Figure 10c,j are the fusion results obtained by the curvelet, DCT_SVD, DTCWT, IFM, LP, MSVD, MWGF, and NSCT methods. Figure 10k is the fusion result obtained by the proposed method. Figure 11 is the local detail magnified regions of Figure 10. Although source images have misregistration, it can be seen from the part marked by the red regions in Figure 11 that the fusion result of the proposed method is very clear at the boundary between the stone lion and the background with fonts. The fusion results of other methods have produced varying degrees of edge blur and artifacts. Obviously, due to the precise detection of the pixel-focus, the proposed method obtains the best fusion results.

#### 3.1.3. Subjective Analysis of More Image Fusion Results

**Figure 12:**
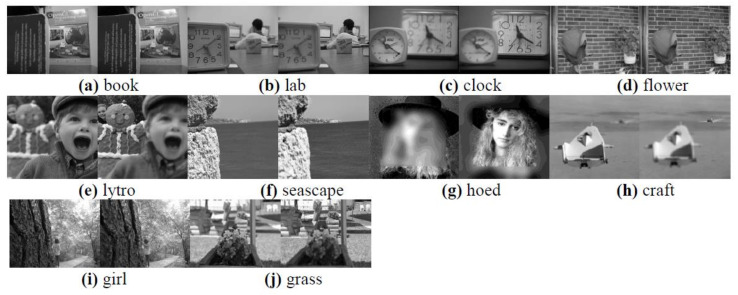
10 pairs of multi-focus source images.

**Figure 13:**
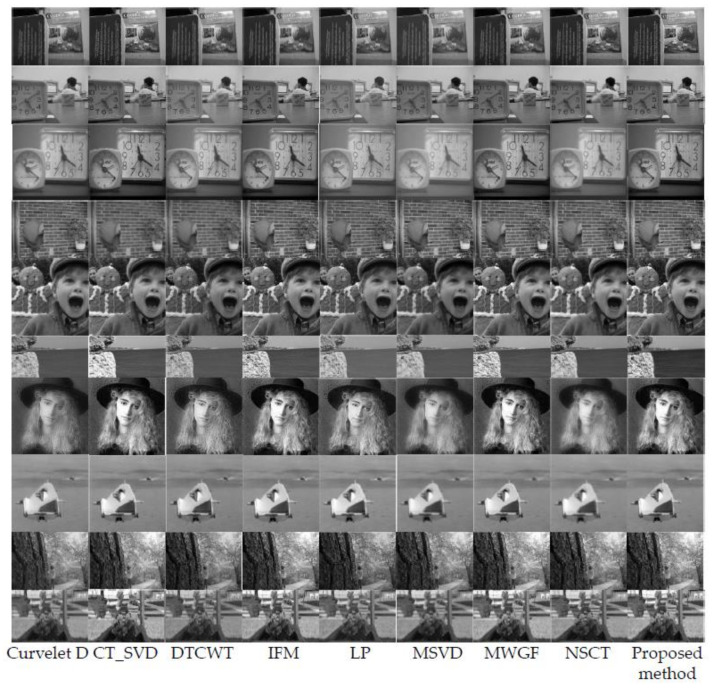
Fusion results of different methods.

In order to further verify the effectiveness of the proposed method, we selected 10 pairs of popular multi-focus source images for comparative experiments, and the source images are shown in Figure 12. Figure 13 shows the fusion results obtained by the proposed method and the other eight methods for comparison. In contrast, the proposed method achieves desirable results in the fusion of 10 pairs of multi-focus images. The proposed method obtains a precise fusion boundary in the fusion results of “book”, “clock”, “flower”, “hoed”, and “lytro”. In the fusion results of “craft”, “grass”, and “seascape” images, clear fusion details are also obtained. In the case where there is a significant difference between the student’s eyes in the “lab” source image and the girl’s body posture in the “girl” source image, the proposed method also obtains a satisfactory fusion result.

#### 3.1.4. Objective Analysis of Fusion Results

The quantitative evaluation of the fusion images has been acknowledged as a challenging task, since, in practice, it lacks of reference images for the source images. In this paper, we selected the edge similarity metric *Q_AB/F_* [25], the normalized mutual information metric *Q_MI_* [1], the phase congruency based fusion metric *Q_PC_* [33], and gradient-based fusion performance metric *Q_G_* [35] to evaluate the fusion results. For all four objective evaluation indicators, the larger the value, the better the fusion results. The highest value in the evaluation is bolded in all tables.

Table 1 shows the objective evaluation values of the fusion results of the nine methods, and the evaluation objects are the “wine” in Figure 5, the “newspaper” in Figure 8, and the “temple” in Figure 10. We can see that the MWGF method has the largest *Q_AB/F_* value in the “newspaper”, and the proposed method fares the best in other evaluation indicators. The method obtains the largest values among the other objective evaluation indicators, which is consistent with the subjective visual effect of the fusion result.

Table 2 shows the *Q_AB/F_* objective evaluation values of the fusion results of 10 pairs of source images with different methods. The proposed method fares the best in other evaluation indicators. The method gets the best fusion results in “book”, “craft”, “flower”, “girl”, “grass”, “lab”, “lytro”, and “hoed”. IFM and MWGF get the best fusion results in “clock” and “seascape”, respectively. This means that, in most cases, the proposed method can incorporate important edge information into the fusion image.

Table 3 shows the *Q_MI_* objective evaluation of the fusion results of 10 pairs of source images with different methods. The proposed method obtains the best fusion results among the nine methods. Although the DCT_SVD method has the highest evaluation values in “flower” and “hoed”, the evaluation value of the proposed method is very close to it, and the variation is less than 0.04.

Table 4 shows the *Q_PC_* objective evaluation values of the fusion results of 10 pairs of source images with different methods. Except for the MWGF method, to obtain the best fusion result in “seascape”, the proposed method has the highest values in other evaluation indicators. This means that the proposed method can well retain important source image feature information of the fused image.

Table 5 shows the *Q_G_* objective evaluation of the fusion results of 10 pairs of source images with different methods. The IFM method achieves the best fusion results in “clock” and “craft”, and the DCT_SVD method in “hoed”. The proposed method fares the best in other evaluation indicators. These mean that the fused image obtained by the proposed method has high sharpness.

**Figure 14:**
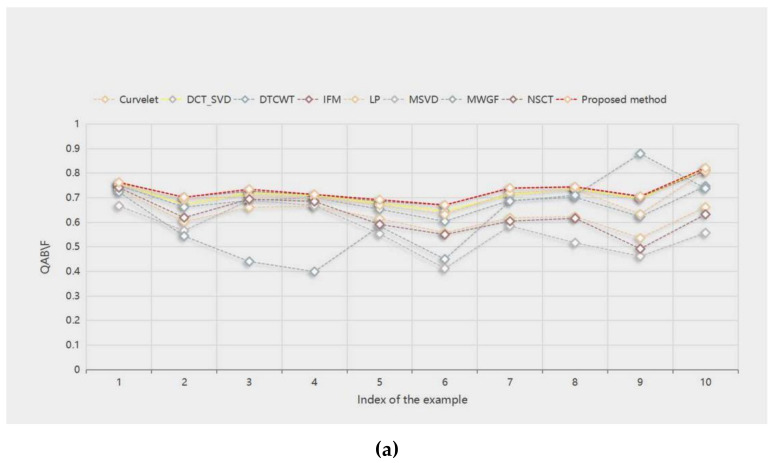

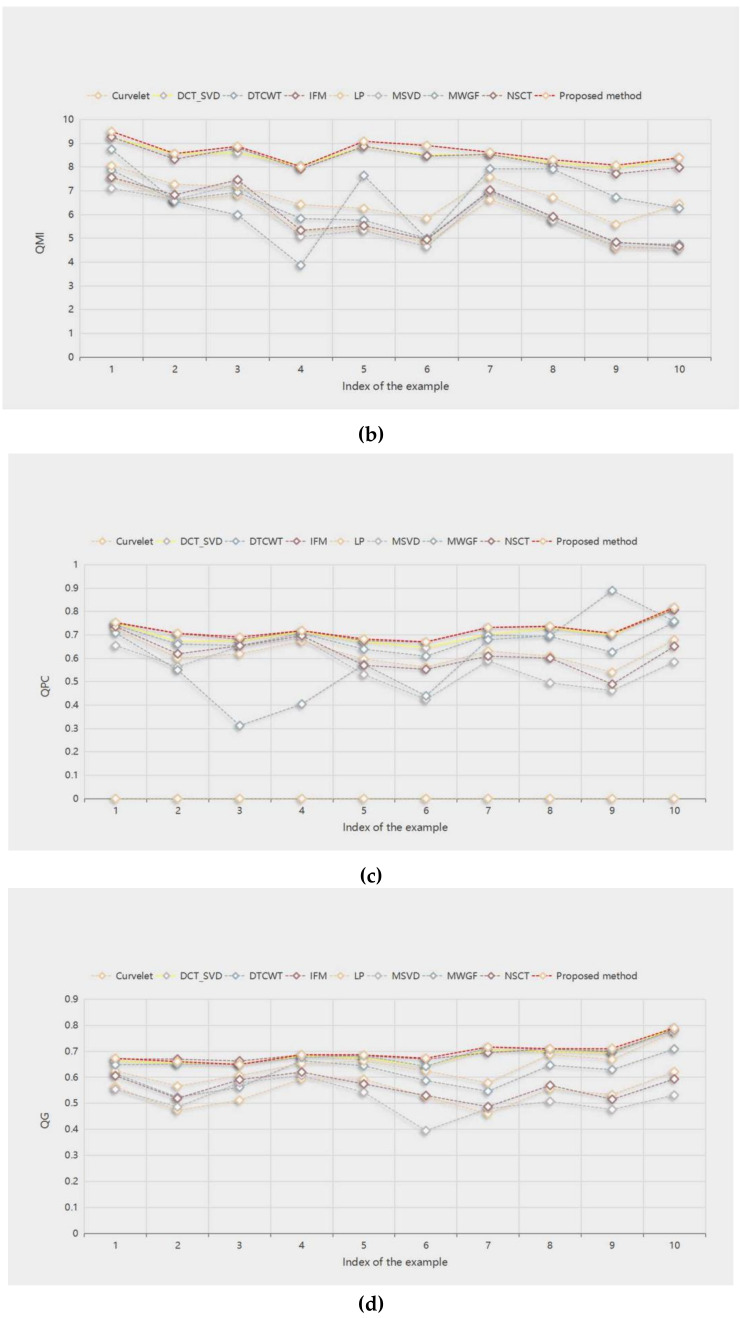
(**a**–**d**) show the score line graphs of the four image evaluation indicators (*Q_AB/F_*, *Q_MI_*, *Q_PC_*, and *Q_G_*) corresponding to Table 2,Table 3,Table 4,Table 5, respectively. In subfigures (**a**–**d**), the horizontal axis represents the image indices ranging from 1 to 10, and the vertical axis represents values of the image evaluation indicators.

Figure 14a–d show the score line graphs of 9 methods on 4 evaluation indicators of 10 pairs of multifocal images, respectively. Obviously, the proposed method fares the best in other evaluation indicators and shows a better scoring trend, compared with other methods. This means that the proposed method fares the best in other evaluation indicators. The method not only suggests better performance in terms of visual perception, but also in quantitative analysis.

#### 3.1.5. Comparison of Computational Efficiency

To compare the computational efficiency, we calculate and list the average fusion time of the nine methods in Table 6. Noticeably, the proposed method takes less fusion time than the IFM and the MWGF methods. The IFM method consumes the most fusion time and the LP method consumes the least fusion time. Comparing the fusion results, it is worthwhile to improve the fusion quality at the cost of the time.

### 3.2. Comparative Analysis of Fusion Results Based on Deep Learning Methods

**Figure 15:**
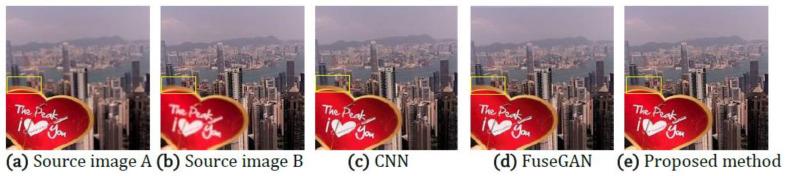
The source images (**a**,**b**) are from the lytro dataset; (**c**) is the fusion result of the CNN; (**d**) is the fusion result of the FuseGAN; (**e**) is the fusion result of the proposed method.

Deep learning, with powerful feature extraction capabilities, has been widely used in multi-focus image fusion. The fusion model obtained through the learning of a large amount of data generalizes well. In order to further verify the effectiveness of the proposed method, it is compared with the deep learning-based multi-focus image fusion methods FuseGAN and CNN proposed in [25]. The comparative experiment in this paper inherits all of the experimental data in [25], including the source images and the fusion results of deep learning methods. The source images in Figure 15 and Figure 17 are from [36] and the lytro dataset [37].

#### 3.2.1. Subjective Analysis of Image Fusion Results

Figure 15 shows the fusion results obtained by the deep learning fusion methods and the proposed fusion method. (a) and (b) are respectively the source images of the front focus and the back focus. (c) and (d) are the fusion results obtained by the CNN and FuseGAN methods. (e) is the fusion result achieved by the proposed method.

**Figure 16:**
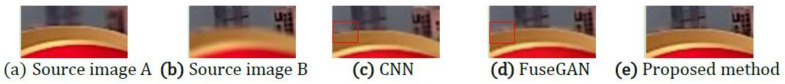
The partial enlarged regions taken from Figure 15a–e.

Figure 16 shows an enlarged region of the local details marked with a yellow frame in Figure 15. In Figure 16, the part marked by the red frame shows that the fused image introduce the blurred edges, which are, respectively, produced by the fusion method of CNN and FuseGAN. The results show that among these methods, the proposed method best preserves the edge information of the source image.

**Figure 17:**
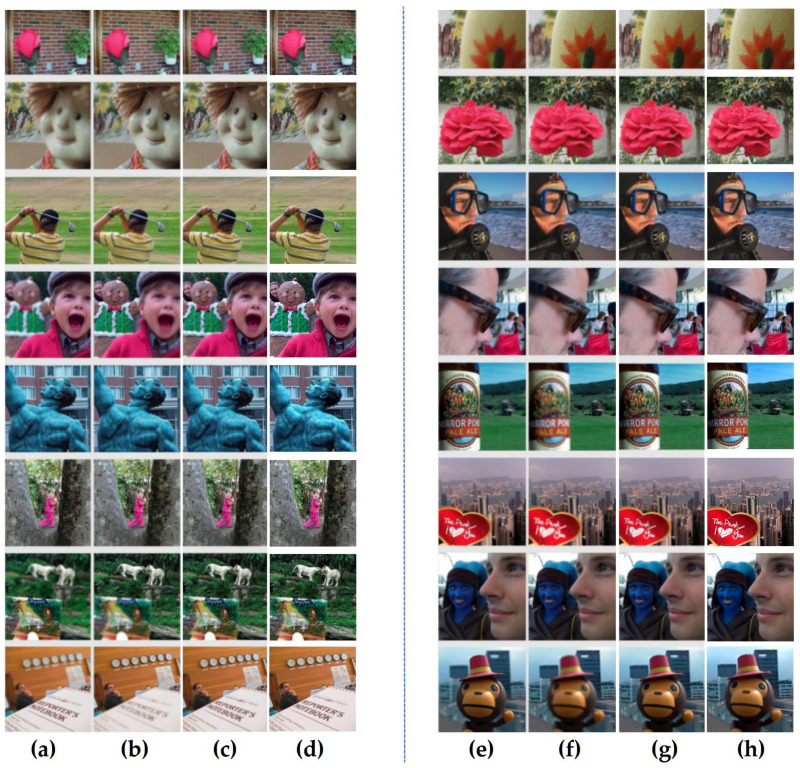
(**a**,**b**,**e**,**f**) show 16 pairs of source images; (**c**,**g**) are the fusion results of the FuseGAN; (**d**,**h**) are the fusion results of the proposed method.

To further verify the effectiveness of the proposed method, 16 pairs of multi-focus source images are selected for comparative experiments. Figure 17 shows the source images and the fusion results. The results reveal that both the proposed method and deep learning method have achieved satisfactory fusion results. Figure 17c,g are the fusion results of the FuseGAN; (d) and (h) are the fusion results achieved by the proposed method.

#### 3.2.2. Objective Analysis of Image Fusion Results

This article selects four evaluation metrics in [25] to evaluate the fusion results, to compare with deep learning methods. They are the edge similarity metric *Q_AB/F_*, the spatial frequency metric *Q_SF_*, the structural similarity metric *Q_Y_*, the feature contrast metric *Q_CB_*. For the above four evaluation metrics, the larger the value, the better the fusion results.

Table 7 shows the mean values of objective evaluations and the average fusion time corresponding to the four metrics when the fusion methods are applied to 29 pairs of source images, with evaluation values of FuseGAN and CNN derived from [25]. The evaluation results show that the proposed method has the best average values in *Q_SF_* and *Q_CB_*. Although the *Q_AB/F_* and *Q_y_* values of the proposed method are smaller than the other two, the difference between them is not greater than 0.015. In summary, the proposed method shows good performance in both visual perception and quantitative analysis.

Table 7 lists the computation efficiency of various methods. As it can be seen, FuseGAN and CNN respectively consume the least and most running times. The running time of the proposed method is slightly longer than that of FuseGAN. Compared with the depth learning method, the proposed method does not need to train the model and parameters in advance and, therefore, is more feasible.

### 3.3. More Analysis

#### 3.3.1. Ablation Research

**Figure 18:**
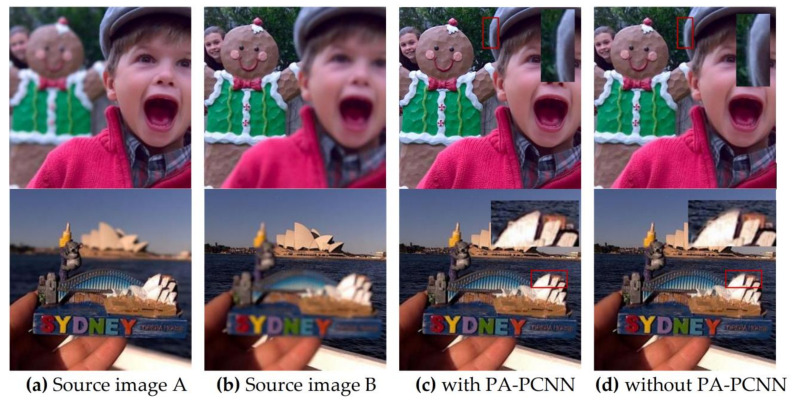
Ablation experiment of the PCNN. (**a**,**b**) are source images; (**c**) results with PCNN; (**d**) results without PCNN.

The parameter-adaptive pulse coupled neural network (PA-PCNN) model can effectively extract image edge and detail information without any training, and all the parameters can be adaptively estimated through the input frequency band. In order to fully investigate the role of PA-PCNN played in the proposed algorithm, the proposed method performs image fusion without it. Specifically, the PA-PCNN fusion strategy is not used in the high-frequency component fusion, but a conventional fusion strategy based directly on the high-frequency decomposition coefficients. This article selects two pairs of images from the lytro dataset for ablation research. In Figure 18c is the fusion result with PA-PCNN, and (d) is the fusion result without PA-PCNN. The upper right corners of (c) and (d) are detailed enlarged views of the area marked with red boxes. In the enlarged detail, the edge of the boy’s hat in (d) and the edge of the Sydney Opera House model have obvious edge blurs, while the same area in (c) is clear. The analyses show that PA-PCNN plays a role in enhancing the fusion effect in the proposed fusion approach.

#### 3.3.2. Series Multi-Focus Image Fusion

**Figure 19:**
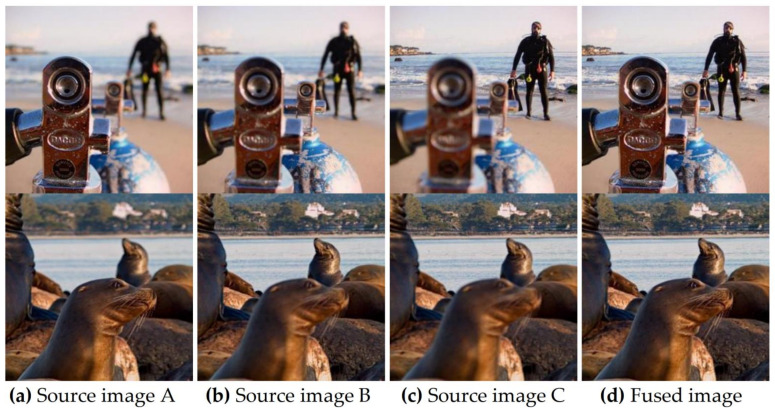
An example of applying the proposed method to fuse three source images. (**a**–**c**) are source images, (**d**) fusion results of the proposed method.

The proposed method can also realize image fusion with more than two multi-focus source images. Figure 19 shows the fusion results of a sequence of three multi-focal sources images. The fusion process of the proposed method goes as follows. Firstly, two of the three source images are selected for fusion; the fused image in the previous step is then fused with the remaining source image to obtain the final fusion images. It can be seen that the focus information of the three source images is well preserved in the final fusion image, with good visual effects.

## 4. Conclusions

In this paper, a multi-focus image fusion method based on multi-scale decomposition with complementary information is proposed. The proposed method achieves the multi-scale double-layer decomposition by constructing an image decomposition scheme with complementary structures and directions. The decomposition method can accurately extract the structure and detailed information of the source images. In order to further ameliorate the fusion quality, different fusion rules are designed according to the characteristics of each decomposition component. In addition, through decomposition and fusion, a decision map with complementary information can be obtained. According to the complementary decision maps, the focus regions and the non-focus regions can be accurately determined, which help solve the fusion problems caused by the anisotropic blur and unregistration of the multi-focus image. The experimental results show that the fusion result of the proposed method is slightly better than the existing methods in terms of image pre-registration and unregistration. Nevertheless, the approach has some limitations and needs refinement. Firstly, in the settings of the method parameters are mainly based on empirical values, and the choice of decomposition scale is one example. The adaptive selection of parameters will be the focus of future research. Moreover, the application of the proposed method to other areas, such as medical image processing and infrared-visible image processing should be part of future exploration.

## Figures and Tables

**Table 1:** The objective assessments of different methods for Figure 5, Figure 8 and Figure 10.

Images	Metrics	Curvelet	DCT_SVD	DTCWT	IFM	LP	MSVD	MWGF	NSCT	Proposed Method
**wine**	*Q_AB/F_*	0.6412	0.6942	0.6752	0.7111	0.6889	0.4467	0.6920	0.6290	**0.7162**
	*Q_MI_*	5.2622	8.4875	5.6663	7.8042	6.5947	4.9732	6.4336	5.4534	**8.6158**
	*Q_PC_*	0.6299	0.6845	0.6649	0.7040	0.0004	0.4206	0.6901	0.6174	**0.7087**
	*Q_G_*	0.4809	0.6501	0.5303	0.6531	0.5679	0.3556	0.6251	0.4950	**0.6682**
**newspaper**	*Q_AB/F_*	0.5244	0.6625	0.6270	0.6659	0.6369	0.3098	**0.6766**	0.4199	0.6751
	*Q_MI_*	1.9036	6.4318	2.2117	5.8831	2.9815	1.7481	5.5151	1.9821	**6.5558**
	*Q_PC_*	0.5043	0.6533	0.6118	0.6568	0.0004	0.2827	0.6639	0.3999	**0.6665**
	*Q_G_*	0.4851	0.6382	0.5878	0.6425	0.6162	0.3349	0.6299	0.4266	**0.6501**
**temple**	*Q_AB/F_*	0.5723	0.7512	0.6715	0.7582	0.7429	0.3474	0.6051	0.5369	**0.7642**
	*Q_MI_*	2.9895	7.2276	3.0351	7.0355	5.1978	3.0224	3.2813	3.1448	**7.3391**
	*Q_PC_*	0.5832	0.7533	0.6795	0.7619	0.0005	0.3658	0.6047	0.5423	**0.7676**
	*Q_G_*	0.5109	0.7146	0.6089	0.7193	0.6891	0.3722	0.7124	0.4922	**0.7203**

**Table 2:** The objective assessment *Q_AB/F_* of different methods for Figure 13.

Images	Curvelet	DCT_SVD	DTCWT	IFM	LP	MSVD	MWGF	NSCT	Proposed Method
**book**	0.7335	0.7594	0.7504	0.7596	0.7532	0.6663	0.7227	0.7408	**0.7628**
**clock**	0.6022	0.6713	0.6618	**0.7025**	0.6920	0.5658	0.5437	0.6190	0.7018
**craft**	0.6605	0.7195	0.6891	0.7267	0.7086	0.6898	0.4401	0.6941	**0.7346**
**flower**	0.6657	0.7098	0.7004	0.7125	0.6985	0.6679	0.3991	0.6848	**0.7133**
**girl**	0.6146	0.6777	0.6528	0.6857	0.6696	0.5530	0.5836	0.5913	**0.6919**
**grass**	0.5574	0.6459	0.6037	0.6694	0.6320	0.4120	0.4496	0.5502	**0.6706**
**lab**	0.6183	0.7116	0.6892	0.7384	0.7194	0.5852	0.6859	0.6046	**0.7394**
**lytro**	0.6233	0.7373	0.7013	0.7428	0.7334	0.5163	0.7101	0.6162	**0.7445**
**seascape**	0.5358	0.6955	0.6231	0.7038	0.6333	0.4614	**0.8794**	0.4920	0.7060
**hoed**	0.6619	0.8207	0.7473	0.8094	0.8074	0.5568	0.7379	0.6323	**0.8212**

**Table 3:** The objective assessment *Q_MI_* of different methods for Figure 13.

Images	Curvelet	DCT_SVD	DTCWT	IFM	LP	MSVD	MWGF	NSCT	Proposed Method
**book**	7.5469	9.2254	7.8636	9.2650	8.0568	7.0990	8.7403	7.5655	**9.4974**
**clock**	6.5747	8.5643	6.6081	8.3289	7.2697	6.6358	6.5600	6.8297	**8.5648**
**craft**	6.8147	8.5981	6.9386	8.8035	7.1822	7.2763	5.9810	7.4597	**8.8691**
**flower**	5.2993	**8.0569**	5.8285	7.9184	6.4240	5.0763	3.8654	5.3363	8.0174
**girl**	5.4226	8.8479	5.7647	8.8677	6.2546	5.3249	7.6408	5.5291	**9.0835**
**grass**	4.8071	8.5131	4.9885	8.4649	5.8314	4.6484	4.9709	4.9466	**8.9043**
**lab**	6.6219	8.5181	6.9902	8.5302	7.5773	6.9382	7.9229	7.0273	**8.6211**
**lytro**	5.7419	8.1906	5.8921	8.0725	6.7115	5.7305	7.9050	5.9048	**8.3023**
**seascape**	4.5815	7.9492	4.8031	7.7184	5.5810	4.6547	6.7174	4.8333	**8.0761**
**hoed**	4.5654	**8.3975**	4.7390	7.9818	6.4462	4.5557	6.2599	4.6683	8.3834

**Table 4:** The objective assessment *Q_PC_* of different methods for Figure 13.

Images	Curvelet	DCT_SVD	DTCWT	IFM	LP	MSVD	MWGF	NSCT	Proposed Method
**book**	0.7254	0.7499	0.7416	0.7503	0.0005	0.6542	0.7081	0.7353	**0.7535**
**clock**	0.6006	0.6718	0.6608	0.7059	0.0005	0.5644	0.5498	0.6190	**0.7067**
**craft**	0.6195	0.6740	0.6547	0.6817	0.0004	0.6505	0.3123	0.6539	**0.6910**
**flower**	0.6722	0.7141	0.7058	0.7179	0.0005	0.6861	0.4042	0.6962	**0.7181**
**girl**	0.5967	0.6712	0.6389	0.6757	0.0004	0.5306	0.5737	0.5702	**0.6827**
**grass**	0.5620	0.6452	0.6090	0.6692	0.0004	0.4238	0.4393	0.5527	**0.6705**
**lab**	0.6307	0.7011	0.6992	0.7314	0.0005	0.5891	0.6794	0.6093	**0.7320**
**lytro**	0.6094	0.7299	0.6928	0.7354	0.0005	0.4946	0.6973	0.6006	**0.7374**
**seascape**	0.5402	0.6994	0.6261	0.7053	0.0004	0.4623	**0.8893**	0.4891	0.7064
**hoed**	0.6792	0.8171	0.7538	0.8074	0.0005	0.5842	0.7582	0.6516	**0.8174**

**Table 5:** The objective assessment *Q_G_* of different methods for Figure 13.

Images	Curvelet	DCT_SVD	DTCWT	IFM	LP	MSVD	MWGF	NSCT	Proposed Method
**book**	0.5636	0.6616	0.6142	0.6704	0.6249	0.5542	0.6482	0.6062	**0.6733**
**clock**	0.4730	0.6538	0.5236	**0.6700**	0.5661	0.4861	0.6509	0.5194	0.6604
**craft**	0.5124	0.6508	0.5619	**0.6629**	0.6070	0.5790	0.6507	0.5911	0.6494
**flower**	0.5936	0.6831	0.6641	0.6863	0.6526	0.6068	0.6770	0.6200	**0.6869**
**girl**	0.5924	0.6737	0.6443	0.6829	0.6628	0.5425	0.6826	0.5744	**0.6859**
**grass**	0.5253	0.6435	0.5871	0.6696	0.6249	0.3950	0.6423	0.5300	**0.6736**
**lab**	0.4604	0.7056	0.5463	0.6946	0.5788	0.4798	0.7153	0.4873	**0.7167**
**lytro**	0.5552	0.6987	0.6465	0.7099	0.6879	0.5074	0.7068	0.5691	**0.7101**
**seascape**	0.5332	0.6945	0.6295	0.6975	0.6687	0.4765	0.7032	0.5154	**0.7116**
**hoed**	0.6216	**0.7912**	0.7090	0.7836	0.7748	0.5316	0.7806	0.5936	0.7896

**Table 6:** Average running time of different fusion methods.

Metric	Curvelet	DCT_SVD	DTCWT	IFM	LP	MSVD	MWGF	NSCT	Proposed Method
**Time** **(Seconds)**	0.9757	1.1396	0.4036	2.2200	**0.3072**	0.3162	1.7036	0.7842	1.4473

**Table 7:** Average objective assessment and running time of different fusion methods.

Metric	*Q_Ab/F_*	*Q_SF_*	*Q_Y_*	*Q_CB_*	Time (Seconds)
**FuseGANe**	**0.7222**	0.0211	**0.9925**	0.8032	**0.53**
**CNN**	0.7177	0.0342	0.9901	0.8001	109.16
**Proposed**	0.7162	**0.007261**	0.9776	**0.8127**	2.85

## Data Availability

Data sharing not applicable.
